# Mutation-rate plasticity and the germline of unicellular organisms

**DOI:** 10.1098/rspb.2019.0128

**Published:** 2019-05-01

**Authors:** Duur K. Aanen, Alfons J. M. Debets

**Affiliations:** Department of Plant Sciences, Laboratory of Genetics, Wageningen University, 6708 PB Wageningen, The Netherlands

**Keywords:** asymmetrical cell division, density-associated mutation-rate plasticity, germline–soma distinction, immortal strand hypothesis, mutation rate, unicellular organisms

## Abstract

The mutation rate is a fundamental factor in evolutionary genetics. Recently, mutation rates were found to be strongly reduced at high density in a wide range of unicellular organisms, prokaryotic and eukaryotic. Independently, cell division was found to become more asymmetrical at increasing density in diverse organisms; some ‘mother’ cells continue dividing, while their ‘offspring’ cells do not divide further. Here, we investigate how this increased asymmetry in cell division at high density can be reconciled with reduced mutation-rate estimates. We calculated the expected number of mutant cells due to replication errors under various modes of segregation of template-DNA strands and copy-DNA strands, both under symmetrical (exponential) and asymmetrical (linear) growth. We show that the observed reduction in the mutation rate at high density can be explained if mother cells preferentially retain the template-DNA strands, since new mutations are then confined to non-dividing daughter cells, thus reducing the spread of mutant cells. Any other inheritance mode results in an *increase* in the number of mutant cells at higher density. The proposed hypothesis that patterns of DNA-strand segregation are density-dependent fundamentally challenges our current understanding of mutation-rate estimates and extends the distinction between germline and soma to unicellular organisms.

## 1. Introduction

Mutation rates are typically minimized, as far as population genetic constraints allow [1]. However, mutation rates can vary, not only between organisms but also with environmental conditions. A recent study identified a completely unexpected kind of mutation-rate plasticity in response to population density [2], which is dependent on quorum sensing [3]. Across a wide range of unicellular organisms, both eukaryotic and prokaryotic, the mutation rate was consistently found to decrease with increasing population density, with up to 23-fold lower mutation rates at high density than at low density. We propose a model that attributes reduced mutation rate at high density to increased asymmetry in mutation acquisition between ‘mother’ cells and ‘offspring’ cells, and discuss recent experimental studies that support this model.

It was long believed that unicellular organisms potentially do not age, thus exhibiting functional immortality. However, the last two decades have seen increasing evidence for asymmetrical cell division leading to differential cell fates, even in organisms with morphologically symmetrical division, such as *Escherichia coli* and fission yeast [4,5]. An asymmetrical cell division results in a senescing ‘mother’ cell and a rejuvenated ‘daughter’ cell, and fecundity of the mother cell decreases with each division as damaged proteins and cell components accumulate. There is increasing evidence that such asymmetries during cell division are not limited to physiological and morphological cell characteristics, but extend to patterns of DNA-strand inheritance, as shown in yeast [6,7] and *E. coli* [8] and various types of stem cells [9,10].

The ‘Immortal Strand Hypothesis' proposes that asymmetries in DNA-strand inheritance reduce the number of mutations in somatic cells [11]. According to this hypothesis, adult stem cells have ‘template-strand co-segregation’ (TSC [9,11]), where the daughter cell maintaining the stem-cell function retains specific ‘master’ templates of the DNA strands of each chromosome (the parental strands [12]) at each division, while the differentiating daughter cell receives the new, ‘copy’ strands. Since most mutations during replication occur in the newly synthesized DNA strands and fewer in the template strands, this asymmetrical distribution reduces the mutation rate in the stem cells [11]. In support of the Immortal Strand Hypothesis, TSC during cell division has been demonstrated in a broad range of organisms [9,10,13–16], although it is not universal for stem cells and alternative hypotheses than reducing the mutation rate have been proposed to explain it [9,17].

Recently, the degree of asymmetry during cell division was found to be higher at high density, in independent studies, for budding yeast [18] and for *E. coli* [19] and other bacteria [20]. Furthermore, for muscle stem cells, asymmetry of strand segregation was found to be increased when stem cells were seeded at higher cell densities [21]. Here, we investigate how those findings of increased asymmetries at high density can be reconciled with reduced estimates of the mutation rate under that condition [2,3]. We show that the observed reduction in the mutation rate at high density can be explained if mother cells preferentially retain the template-DNA strands, since new mutations are then confined to non-dividing daughter cells, thus reducing the spread of mutant cells.

## 2. Methods

### (a) The average number of mutations due to copy errors during linear growth

**Figure 1.**
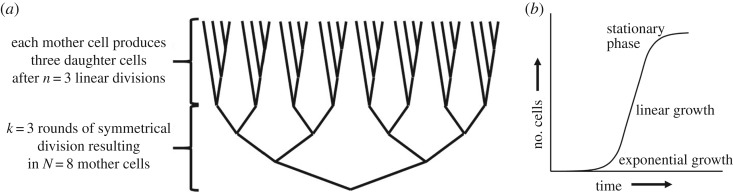
Schematic illustration of the shift in growth from symmetrical and exponential to asymmetrical and linear at high density. (*a*) Initially, when nutrition is not limiting, exponential growth occurs, resulting in *N ‘*mother’ cells. At higher density, those mother cells act as stem-cell lineages, continuing to bud off ‘offspring’ cells in a linear fashion. Those offspring cells stop dividing and become quiescent. (*b*) Graph sketching the number of cells over time when growth shifts from exponential to linear.

We derive an expression for the expected number of mutant cells during linear growth, as a function of the probability that the mother cell inherits the template-DNA strands, following similar analyses for stem cells [10]. We consider mutations due to copy error, the most common class of mutations [22], so any mutations occur in the copied strand, and not in the template strand. We consider a culture of unicellular organisms that starts growing exponentially, and then shifts to linear growth (figure 1*a*). At the end of the exponential growth phase, there will be *N* cells. We assume that those cells all become mother cells that start dividing in a linear fashion, meaning that they bud off a finite number of daughter cells that do not divide further.

Each mother cell divides *n* times during a time interval Δ*t*. With each division, a mother cell non-randomly segregates DNA strands with a probability *p*. If *p* = 1, the mother cell always retains the template strands, while *p* = 0.5 implies random strand segregation (RSS). We assume that the probability *p* is the same for all mother cells. The copy strands acquire on average *μL* new mutations, where *μ* is the mutation rate per base pair per cell division and *L* is the genome size, and we assume that mutations are neutral.

For *n* cell divisions, the probability that a mother cell inherits template-DNA strands *k* times is binomially distributed (*k* successes in *n* draws given a success probability of *p*)

2.1 $$P(k,n,p)=\left(\begin{array}{c} n\\ k \end{array}\right)p^{k}{\left(1-p\right)}^{n-k}.$$

This implies that on average in *E*[*k*,*n*,*p*] = *np* cell divisions, the mother cell receives the template strands and thus does not receive additional mutations. Conversely, on average in *n*(1 − *p*) cell divisions, the mother cell receives the copied strands, thus receiving *μL* additional mutations. Therefore, the average number of mutations a mother cell carries (the so-called mutational burden $\tilde{\mu }$) is

2.2 $$\tilde{\mu }=n(1-p)\mu L.$$

The variance of the mutational burden $\sigma^{2}$ is given by [10]

2.3 $$\sigma^{2}=n(1-p)\mu L+n{\left(\mu L\right)}^{2}p(1-p).$$

With these expressions, we can quantify the change of the mutational burden and the change of the variance of the mutational burden per mother cell after a number of Δn divisions

2.4 $$\frac{\Delta \tilde{\mu }}{\Delta n}=(1-p)\mu L$$

and

2.5 $$\frac{\Delta \sigma^{2}}{\Delta n}=(1-p)\mu L+{\left(\mu L\right)}^{2}p(1-p)$$

### (b) Calculating the number of mutant cells in a culture

Since microorganisms generally have small genomes, it is hard to measure the increase in the number of mutations in individual mother and daughter cells, and the change in variance in mutation burden among mother cells. However, since the genome size is small, and the number of cell divisions is limited, it is reasonable to assume that a cell acquires maximally a single mutation. With this assumption, we can derive an expression for the increase in the number of mutant cells in a population, and from that *p*, as shown below.

For a culture of size *N* when entering the linear growth phase, the total number of additional mutations (*m*) acquired during *n* linear divisions can be calculated. For each cell division, there is a chance *p* that the mother cell receives the template strands, and a daughter cell will then acquire *μL* new mutations. In a population of size *N* mother cells, undergoing *n* divisions, this yields a total of *npμLN* new mutant cells. The remaining fraction of cell divisions (1 − *p*) will yield a higher number of mutant cells, since a mutation acquired by a mother cell will be passed on to additional daughter cells produced in subsequent divisions of that mother cell. However, this depends on the moment the mother cell acquires the mutation: early acquisition yields many mutant cells, late acquisition few. The expected number of new mutant cells due to mutations in mother cells is

$$\begin{array}{rl} \overset{n}{\underset{i=1}{\sum }}(1-p)iN\mu L & =(1-p)nN\mu L+(1-p)(n-1)N\mu L\\ & \;+\cdots (1-p)N\mu L\\ & =(1-p)N\mu Ln\left(\frac{n+1}{2}\right). \end{array}$$

The total number of new mutant cells, Δ*m*, in a population of size *N* mother cells, undergoing *n* divisions is

2.6 $$\Delta m=npN\mu L+(1-p)N\mu Ln\left(\frac{n+1}{2}\right)=N\mu Ln\left((1-p)\left(\frac{n+1}{2}\right)+p\right).$$

*p* can be calculated by determining the increase in the number of mutant cells between samples taken at two time points in the linear growth phase, provided we have estimates of *N*, μL, and the number of divisions separating the two samples (*n*). The latter number is equal to the increase in population size.

As shown above, variance in mutational burden among mother cells increases over time. Variance among cultures in mutation burden of the average mother cell corresponds to the variance of a mean, which is the variance divided by the sample size. Thus, the variance among cultures is equal to the variance in mutational burden among mother cells divided by the number of mother cells *N*

2.7 $$\sigma_{\mathrm{among}\;\mathrm{cultures}}^{2}=\frac{n(1-p)\mu L+n{\left(\mu L\right)}^{2}p(1-p)}{N}\;.\;\;\;\;\;$$

And, the change in variance among cultures after *n* divisions is

2.8 $$\frac{\Delta \sigma_{\mathrm{among}\;\mathrm{cultures}}^{2}}{\Delta n}=\frac{\left(1-p)\mu L+{\left(\mu L\right)}^{2}p(1-p\right)}{N}.$$

Considering that *N* will be large at the end of exponential growth, variance among cultures will be very low.

### (c) The number of mutant cells due to copy errors during exponential growth

To compare the increase in the expected number of mutant cells in the case of symmetrical division and exponential growth, we first calculate the number of rounds of cell divisions *k* required for an *n*-fold increase in population size under exponential growth, which is

$$k=\frac{\ln(n)}{\ln(2)}.$$

The expected number of additional mutant cells Δ*m*, after *k* rounds of cell division, starting with a population of size *N* is

2.9 $$\Delta m=N\mu L\;*\;\frac{k2^{k}}{2}.\;$$

We divide by a factor 2, since we only consider copy errors, so a mutational event leads to a mutant cell in only one of the two daughter cells formed.

## 3. Results

Consider a culture of unicellular organisms grown under high nutrition conditions (figure 1). Initially, when nutrition is not limiting, growth will be maximal and exponential [23]. When nutrition becomes limiting, growth will increasingly become non-exponential (figure 1*b*). As has been shown for yeast and bacteria at high density, mother cells start to act like stem-cell lineages that continue budding off rejuvenated offspring cells for their entire replicative lifespan or for the remainder of it, while the rejuvenated offspring cells are quiescent and do not divide further [20,24] (figure 1*b*). At low nutrition, the transition to non-exponential growth is less strictly associated with differentiation between mother cells and rejuvenated offspring cells [18,19,21].

**Figure 2.**
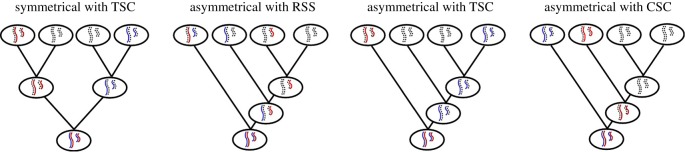
A comparison between symmetrical cell division with TSC (left) and asymmetrical cell division with three forms of DNA-strand inheritance: RSS (*p* = 0.5; centre left), TSC (*p* = 1; centre right), and CSC (*p* = 0; right). Following DNA replication, an asymmetrical cell division results in two daughters, one of which becomes a new mother, and the other of which is a rejuvenated cell that stops dividing. According to the ‘Immortal Strand Hypothesis', the sister chromatids containing the older strands (blue non-dashed) are retained in the continually dividing mother cell while the chromatids containing the copy strands (dashed) are inherited by the daughter cells. Since the segregation pattern does not influence the number of mutant cells when cell division is symmetrical, symmetrical cell division is only drawn for one type of strand inheritance.

We consider the effect of DNA-strand inheritance on the expected number of mutant cells, when growth shifts from exponential to linear. For symmetrical growth, this number does not depend on inheritance patterns of DNA-template and copy strands, since all cells continue dividing. However, for asymmetrical growth, the number of mutant cells is influenced by the inheritance pattern of DNA strands. Equation (2.6) shows that the number of mutant cells decreases with increasing *p*, the probability that the mother cell inherits the template-DNA strands. We consider three specific values of *p* (figure 2): (i) *p* = 0, meaning that the copied strands are always inherited by the mother cell (copy-strand co-segregation; CSC), (ii) *p* = 1, where the template strands are always inherited by the mother cell (template-strand co-segregation; TSC), and (iii) *p* = 0.5, where template and copied strands are randomly distributed over mother and daughter cells (RSS). We compare those three cases with the number of mutant cells expected for symmetrical division.

**Figure 3.**
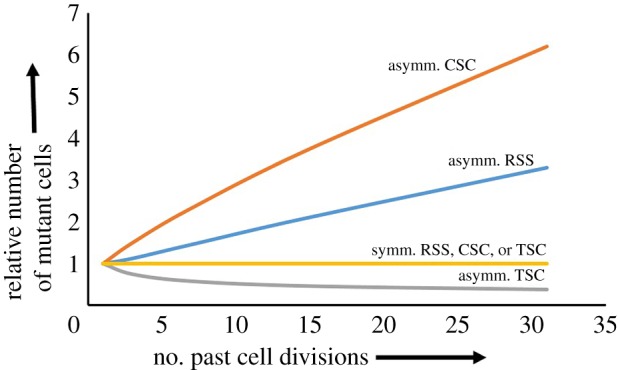
The expected number of mutant cells under various models of division and template-strand segregation relative to that expected for symmetrical (exponential) growth, as a function of the number of past cell divisions. Orange line: symmetrical cell division (with CSC, TSC, or RSS); red line: asymmetrical cell division with CSC (*p* = 0); blue line: asymmetrical cell division with RSS (*p* = 0.5); grey line: asymmetrical division with TSC (*p* = 1). The difference in the expected number of mutant cells under asymmetrical division relative to that for symmetrical growth increases with the number of cell divisions. Asymmetrical division with TSC yields the lowest expected number of mutant cells, and the relative difference with other types of cell division and/or DNA-strand inheritance increases with the number of cell divisions.

For *p* = 1 (TSC), equation (2.6) simplifies to: Δm=NμLn, and for *p* = 0 (CSC) to Δm=NμLn(n+1/2). For *p* = 0.5 (RSS), equation (2.6) simplifies to Δm=NμLn((n+1/4)+0.5). For symmetrical division, $\Delta m=N\mu L\;*\;(k2^{k}/2)$, where the rounds of cell division *k* corresponding to an increase in population size with linear division *n*, can be calculated as k=ln⁡(n)/ln⁡(2). To simplify comparison of those four cases of cell division and DNA-strand inheritance, we determined the expected number of mutant cells after *n* divisions when the common factor between all four formulas *NμL* = 1 (table 1). We also determined the expected number of mutant cells relative to that expected for symmetrical growth, for those four cases (figure 3).

**Table 1.** The expected numbers of mutant cells during the formation of n cells under symmetrical and asymmetrical cell division, and with either RSS, CSC, or TSC.

symmetrical cell division						asymmetrical cell division							
				expected no. mutant cells				RSS		CSC		TSC	
								expected no. mutant cells					
rounds of cell division	no. cells	no. past cell divisions	branch length in tree	divided by NμL	relative to symmetrical	no. cells	no. past cell divisions	divided by NμL	relative to symmetrical	divided by NμL	relative to symmetrical	divided by NμL	relative to symmetrical
0	1	0	0	0	n.a.	1	0	0	n.a.	0	n.a.	0	n.a.
1	2	1	2	1	1	2	1	1	1	1	1	1	1
2	4	3	6	4	1	4	3	4.5	1.13	6	1.5	3	0.75
3	8	7	14	12	1	8	7	17.5	1.46	28	2.33	7	0.58
4	16	15	30	32	1	16	15	67.5	2.11	120	3.75	15	0.47
5	32	31	62	80	1	32	31	263.5	3.29	496	6.2	31	0.39
6	64	63	126	192	1	64	63	1039.5	5.41	2016	10.5	63	0.33
7	128	127	254	448	1	128	127	4127.5	9.21	8128	18.14	127	0.28
8	256	255	510	1024	1	256	255	16 447.5	16.06	32 640	31.88	255	0.25
9	512	511	1022	2304	1	512	511	65 663.5	28.50	130 816	56.78	511	0.22
10	1024	1023	2046	5120	1	1024	1023	262 399.5	51.25	523 776	102.3	1023	0.20

With RSS (*p* = 0.5), the expected number of mutant cells exceeds that of symmetrical division, and the difference increases with increasing numbers of cell divisions. The expected number of mutant cells is even higher if the daughter cell preferentially inherits the template strand (CSC; *p* = 0). By contrast, if mother cells preferentially inherit the template strands (template-strand co-segregation; *p* = 1), the expected number of mutant cells falls behind that of symmetrical division, and this difference increases with increasing numbers of cell divisions.

For asymmetrical growth, the inheritance pattern thus has a strong effect on the expected number of mutant cells. Consider asymmetrical division of a mother cell that can still bud off 31 daughter cells [25]. This results in a 32-fold increase in population size, corresponding to five rounds of symmetrical division. CSC would then be expected to yield 6.2 times more mutant cells than symmetrical cell division (irrespectively of the pattern of template and copy-strand segregation), and RSS 3.3 times more. Conversely, TSC would yield 2.6 times fewer mutant cells than symmetrical division (table 1; see Methods for the calculation). For yeast, where the maximal replicative lifespan has been determined at some 30 cell divisions [25], a 2.6-fold reduction in the expected number of mutant cells compared to symmetrical division leading to the same number of cells seems the maximum.

We calculated the minimum value of the asymmetry in template-strand inheritance *p*, above which linear growth yields fewer mutant cells than exponential growth, for different values of *n*. To do so, we equated formula (2.6) and formula (2.9). For *n* = 31 linear divisions (corresponding to *k* = 5 rounds of symmetrical division), this yields *p* = 0.89 and for *n* = 15 (corresponding to *k* = 4 rounds of symmetrical division), *p* = 0.84. These results show that DNA-template-strand inheritance needs to be strongly asymmetrical for a reduction in the expected number of mutant cells when growth shifts from exponential to linear.

## 4. Discussion

Our results show that the empirical finding of a reduced mutation rate at high density can be reconciled with increased asymmetry in cell division under that condition if mother cells, which continue to divide, have a higher probability of maintaining the template strands. Asymmetrical cell division with complete TSC (*p* = 1) can account for a significant reduction in mutation-rate estimates, although not sufficient to fully explain the density-dependent mutation-rate plasticity recently reported [2,3]. However, there is another catch when growth shifts from exponential to linear. Estimates of the mutation rate assume exponential growth [26]. The fluctuation test takes into account the probability that a mutation occurs at an early growth stage, in which case a large proportion of the population will have the mutation (a so-called jackpot). The model proposed here, with linear growth by division from a stem-cell-like mother that tends to retain the template strands will never yield a ‘jackpot’, since mutations in the non-exponential phase always occur in terminal branches. This implies that the mutation rate will be systematically underestimated, which may account for the remaining difference. Furthermore, if asymmetrical growth occurs in the later stages of both low-density and high-density conditions, but TSC only at high density, the difference in mutation rate between low and high density will further increase (figure 3).

To measure the degree of DNA-template-strand inheritance during linear growth (*p*), the number of mutant cells at different time points in the linear growth phase needs to be measured to calculate *p*. Equation (2.7) predicts that variance among cultures in the number of mutations acquired during the linear growth phase is low, so in principle this would not require many replicate estimations. In order to judge the proposed model and its generality, several assumptions and predictions need to be tested. First, more detailed insight in the later stages of growth of microbial organisms and its dependence on density is needed [4,5,27]. For yeast, a transition from exponential to linear growth has been established [24]. Growth is exponential during the anaerobic phase when sugar is fermented, but linear in the aerobic phase when ethanol is used as a carbon source via respiration [28,29]. The daughter cells formed in the linear phase are in a quiescent state and do not divide further. Also in *E. coli*, cultures at higher densities contain a subpopulation of quiescent cells and the proportion of quiescent cells increases with cell density [30]. In the bacterial species *Dinoroseobacter shibae*, at high density, bacteria switch from exponential to linear growth and quorum sensing regulates this switch [20]. Second, the relationship between cell density and the degree of asymmetry in DNA-template-strand inheritance needs to be established. While the degree of asymmetry in cell division has been demonstrated to be density dependent for multiple organisms [18–20], increased asymmetry of DNA-strand inheritance has only been demonstrated in one case, for muscle stem cells grown *in vitro* [21]. Finally, our model assumes a sharp transition from exponential to linear growth. More realistic models may investigate gradual changes from exponential to linear growth and also the transition from linear growth to the stationary phase [28].

The apparent universality of density-associated mutation-rate plasticity begs for a general mechanism. Given the independent evidence for a link between the degree of asymmetrical cell division and density in widely divergent organisms as bacteria [19], single-celled eukaryotes [18], and stem cells of multicellular eukaryotes [21], it seems plausible that this mechanism is based on asymmetrical cell division. The model proposed here is best supported for yeast. In yeast, at high density, a larger fraction of the cells become quiescent, being arrested in the *G*_0_ phase of the cell cycle [18], and consisting almost exclusively of rejuvenated quiescent daughter cells with a high capacity to grow when conditions improve [24]. The remaining cells are heterogeneous and show senescence. In support of a role for TSC, in yeast, asymmetries in kinetochore inheritance have been shown [6], and one study found support for asymmetrical strand segregation [7], although another study did not [31]. However, the latter study used a low population density, which may account for this difference.

It seems paradoxical that the senescing cell retains the template-DNA strands, and thus acquires the fewest mutations, while the rejuvenated offspring cells receive the copied strands, and thus any mutant cells. However, as explained above, this inheritance pattern reduces the number of mutants among the rejuvenated cells. Perhaps the strongest argument in favour of the model proposed in this article is that the mutation rate will be strongly *increased* and not decreased if DNA strands were inherited randomly when cell division becomes asymmetrical. Even for the production of 16 rejuvenated cells by a mother cell, asymmetrical cell division with RSS would yield 4.5 times more mutant cells than asymmetrical cell division with TSC, and still over two times more than symmetrical division (figure 3 and table 1). The finding of, on the one hand, a reduction in the mutation rate at high density [2,3] and, on the other hand, an increase in asymmetrical division at high density [18,19,21], therefore, makes it plausible that template-strand co-segregation occurs. However, direct evidence for our model remains to be provided. Recent improvements in the detection of mutations in single cells may make it feasible to test our hypothesis directly [32,33]. An intriguing question is whether our model also applies to density-associated mutation-rate plasticity found in viruses [2]. Since viruses are dependent on their host for genome replication, in the experiments used to measure the mutation rates at various densities, virus density may correspond to host density, in which case our model may also apply to viral replication. It has been proposed that the mutation rate of RNA viruses may also depend on their replication mode, either by exponential replication where copy strands are copied or linear replication where template strands are used for replication only [34].

The plasticity in mutation rate in response to population density implies that numbers of mutational events per space and time vary much less with final population size than expected from a fixed mutation rate per cell division. In other words, the total number of cells with mutations occurring in a high-density and a low-density culture of unicellular organisms are more similar than expected based on the number of cell divisions that have occurred. This buffered number of mutant cells per space and time fits remarkably well in an emerging picture that the mutation rate of organisms is reduced by specific aspects of their growth mode, not only for vertebrate animals, which set aside germ cells early in development, but also for organisms that do not. For example, taller, long-lived plants have been found to have lower rates of molecular evolution per unit time than small plants, implying that the mutation rates per generation are more similar [35]. In plant meristems, the stem cells from which reproductive organs will develop undergo a minimal number of divisions during plant growth [36]. Also, the number of cell divisions separating axilliary meristems from the central meristem is minimized [37]. Similarly, in a fungus with an estimated age of more than 1500 years, the number of mutations was much lower than expected, presumably due to an unknown mechanism to reduce the number of mitotic divisions of cells at the growth front [38,39]. In ciliates, a transcriptionally silent germline nucleus is present, whose mutation rate per cell division is more than an order of magnitude lower than that of other eukaryotes, but, converted to a per-sexual generation mutation rate, is remarkably similar to that of multicellular eukaryotes with a similar genome size [40].

The realization that unicellular organisms also have mechanisms to reduce the mutation rate makes the germline–soma distinction more general than once believed. Weismann [41] was the first to distinguish an immortal germline from a disposable soma and argued that variations within individuals cannot be transmitted to the germline. Buss [42] challenged Weismann's doctrine, noticing that an early germline sequestration as seen in vertebrates is rare among multicellular organisms. The recent findings discussed in this paper, however, revive part of Weismann's doctrine. A picture emerges that germline sequestration is not limited to some animals, but also occurs in plants, fungi, and even unicellular organisms, although the timing of sequestration may vary between organism groups and with ecological conditions such as population density.

## Data accessibility

This article has no additional data.
